# Time Course of Root Axis Elongation and Lateral Root Formation in Perennial Ryegrass (*Lolium perenne* L.)

**DOI:** 10.3390/plants10081677

**Published:** 2021-08-15

**Authors:** Arif Hasan Khan Robin, Louis John Irving, Jim Crush, Hans Schnyder, Fernando Alfredo Lattanzi, Cory Matthew

**Affiliations:** 1 Department of Genetics and Plant Breeding, Bangladesh Agricultural University, Mymensingh 02202, Bangladesh; 2 School of Agriculture and Environment PN433, Massey University, Private Bag11-222, Palmerston North 4442, New Zealand; c.matthew@massey.ac.nz; 3 Graduate School of Life and Environmental Sciences, University of Tsukuba, Tsukuba 305-8577, Japan; irving.louis.fb@u.tsukuba.ac.jp; 4 AgResearch, Ruakura Research Centre, PB 3123, Hamilton 3240, New Zealand; jim.crush@agresearch.co.nz; 5 Lehrstuhl für Grünlandlehre, Technische Universität München, 85354 Freising, Germany; schnyder@wzw.tum.de or; 6 Programa de Pasturas y Forrajes, Instituto Nacional de Investigación Agropecuaria (INIA), Estación Experimental INIA La Estanzuela, Ruta 50 km 11, 39173 Colonia, Uruguay; flattanzi@inia.org.uy

**Keywords:** *Lolium perenne*, root development, root elongation, lateral roots, root branching, root dry weight, root surface area, root volume, phytomer

## Abstract

Grasses have a segmental morphology. Compared to leaf development, data on root development at the phytomer level are scarce. Leaf appearance interval was recorded over time to allow inference about the age of segmental sites that later form roots. Hydroponically grown *Lolium perenne* cv. Aberdart tillers were studied in both spring and autumn in increasing and decreasing day length conditions, respectively, and dissected to define the development status of roots of known age on successive phytomers basipetally on the tiller axis. Over a 90-day observation period spring and autumn tillers produced 10.4 and 18.1 root bearing phytomers (Pr), respectively. Four stages of root development were identified: (0) main axis elongation (~0–10 days), (1) primary branching (~10–18 days), (2) secondary branching (~18–25 days), and (3) tertiary and quaternary branching without further increase in root dry weight. The individual spring roots achieved significantly greater dry weight (35%) than autumn roots, and a mechanism for seasonal shift in substrate supply to roots is proposed. Our data define a root turnover pattern likely also occurring in field swards and provide insight for modelling the turnover of grass root systems for developing nutrient efficient or stress tolerant ryegrass swards.

## 1. Introduction

**Figure 1:**
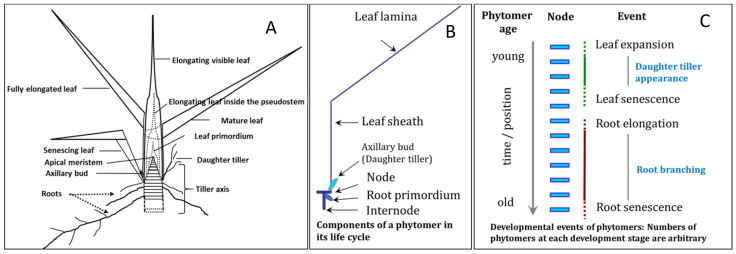
Developmental stages of phytomers in a vegetative grass tiller. In diagram (**A**) phytomers are represented as stacked discs within the tiller axis. Phytomer development progresses with phytomer age moving basipetally down the axis from the apical meristem, with the final event in the phytomer life cycle being root elongation. Leaf developmental stages have been drawn taking data from Yang et al. [3]. The diagram has been drawn following Matthew et al. [7]. Diagram (**B**) represents the morphological components of a typical vegetative-tiller phytomer. Diagram (**C**) illustrates the spatial separation on the tiller axis and temporal separation for a particular phytomer, of leaf and root developmental events.

Grass tillers are composed of recurring segmental units known as phytomers. Over their life cycle, phytomers undergo a series of developmental changes following their differentiation at the apical meristem [1,2,3], including the formation, maturation, and subsequent senescence of a leaf, the possible release within a limited time window of a leaf axillary bud to form a daughter tiller, possible internode elongation, and usually the formation of roots within a few days of leaf senescence (Figure 1). In a grass tiller, the development of leaves exhibits a coordination between adjacent phytomers (e.g., [4,5,6]). Developmental events such as leaf initiation, ligule formation, and the cessation of cell division at the leaf sheaths are often recorded in phyllochron time units—the interval between the appearance of successive leaf tips from the sheath of the preceding leaf, and reported in days or growing degree days (Figure 1, [4]).

In comparison to leaf development, little has been reported on root developmental succession along the tiller axis. Early field studies typically reported an annual pattern of root replacement under grass swards without clarifying root formation and developmental activity at the tiller axis (e.g., [8,9,10]). Subsequently, some reports have documented the positional placement of individual roots on the tiller axis [3,11,12]. Matthew et al. [13] proposed the development of sward mass flow equations based on summation of events at the tiller axis level. From a whole sward perspective, when new root deposition in perennial ryegrass dominant swards was isolated from the bulk root mass by an ‘ingrowth core’ technique, the data indicated continuous new root deposition with a mass flow of about 15% of leaf formation, and with peaks in spring and following autumn rain [14].

Little previous research has examined the developmental morphology of individual roots on the tiller axis. It is axiomatic that so long as there is a lack of data on root developmental activity and function over the life-span of root-bearing phytomers on the tiller axis, it will be difficult to formulate breeding criteria for enhancement of root contribution to forage grass performance, or even understand which root system traits might be beneficial. A range of methodologies, such as minirhizotrons [15,16,17] and core-break techniques [16,18] have been devised by researchers interested in quantifying root development and function, but each of the various techniques has limitations [19]. Here, we address the knowledge gap in the root development domain through a new technique not previously used before, to our knowledge. In our study, ryegrass tillers were grown hydroponically from excised tiller ramets, and their leaf appearance and senescence recorded for approximately 90 d, meaning that for phytomers on the tiller axis below the oldest leaf, the age of each phytomer could be inferred from its leaf-appearance date. By destructive harvest and careful separation of individual roots, it was possible to isolate an age-series of roots for each of the dissected tillers, with roots from successive phytomers one phyllochron older than roots from the last. In each developmental series, individual phytomers bore variable numbers of roots (there were 0–6, but typically two roots per phytomer; the sites for root appearance were not random, but were usually located at 45° and 135° around the circumference, taking the tiller bud at the node as 0°). Two experiments were conducted using the same hydroponic equipment in the same glasshouse under contrasting conditions of increasing temperature and day length in spring and decreasing temperature and day length in autumn. A technique description and some preliminary data from this work for the New Zealand ryegrass cultivar Alto, were previously included in a conference presentation [20], which reported progressive, coordinated root development beginning with root initiation at or near the senescing leaf, continuing for a time span represented by approximately seven to nine adjacent phytomers. We subsequently used root mass data from this experiment, together with assumptions about substrate (photosynthate) supply and respiration rates, to model the carbon economy of individual roots through their development cycle [21]. Our model suggested that the shoot C supply was shared relatively equally between phytomer positions; with the C-costs of maintenance respiration dominating the C budget of older roots, while smaller young roots had lower maintenance needs and could invest a greater proportion of C in new growth. Seasonally driven shifts in plant photosynthesis and C balance were hypothesized to explain root system growth in spring, and the death of primarily older roots in the autumn. In addition, Matthew et al. [7,13] have noted that because root production at a phytomer follows after a leaf has grown and senesced, the rhizochron is expected to conform to the phyllochron a few leaf appearance intervals earlier, meaning that as the phyllochron changes seasonally with temperature, the rhizochron:phyllochron ratio will not be constant but will also change seasonally. This variation in rhizochron:phyllochron ratio, if not neutralized by other compensatory factors could potentially change C supply to individual roots on a seasonal basis, with a theoretical increase in supply to individual roots in the root system of spring tillers and decrease in supply to individual roots in the root system of autumn tillers. In part this effect could be mediated by comparatively fewer roots being fed per leaf in spring and comparatively more roots per leaf in autumn. Gathering evidence for this postulated seasonal shift in C supply to roots would not be straightforward, but a good starting point would be a measure that integrates effects of plant processes over time, such as mature root dry weight. Hence, our experiment included this measurement.

In this paper, we report data for the United Kingdom-bred perennial ryegrass cultivar Aberdart. There were two research objectives: (i) To present descriptive data on root development over time, including root dry weight (RDW), root axis length (RAL), number of roots per phytomer (Rp), and a WinRhizo analysis of an age-series of roots to document the morphological development and branching process from root appearance to around 80 days of age and formulate a synthesis of the root development process. (ii) To compare data collected in spring and autumn for any evidence of seasonal differences in numbers of new roots formed per new leaf, and differences in mature root dry weight that might be attributable to seasonal difference in the rhizochron:phyllochron ratio.

## 2. Results

An inventory of the data collected, the numbers of plants or roots involved in each measurement and the analytical procedures employed, appears in Table S1 and Supplementary Data File S1.

### 2.1. Plant Environment and Plant Development

In spring, 10-day mean temperature increased from 7.6 °C at the start of the experiment to 14.7 °C in the final 10 days, while in autumn 10-day mean temperature fell from 21.4 °C in the first 10 days (with maxima > 30 °C on 6 days) to 16.6 °C in the closing 10 days. The average phyllochron length was 9.22 and 4.44 d in spring and autumn, respectively. As a result, spring tillers had on average 10.4 ± 0.17 live root bearing phytomers compared with 18.1 ± 0.53 in autumn plants (*p* < 0.001) of similar age. The leaf elongation rate (LER) rose with temperature in spring from 1.4 to 2.5 mm °C.d^−1^ over the course of the experiment. In autumn LER rose with falling temperatures over the first 30 days of the experiment from 2.0 to 2.7 mm °C.d^−1^, then as temperatures fell further LER decreased to 1.7 mm °C.d^−1^ at the end of the experiment. Tillers developed to a larger size in the autumn experiment than in the spring experiment. Leaf dry weight per tiller was more than 3× higher and number of live leaves per tiller 1.5-fold higher in autumn tillers than in spring tillers (Table 1, [19]).

### 2.2. Root Dry Weight, Main Axis Length and Number of Roots per Phytomer

Root dry weight from individual phytomers (RDWp) of spring tillers was less than half of that in autumn tillers (Table S2, *p* = 0.006 in repeat measures ANOVA), but this should be interpreted in conjunction with the more than three-fold greater leaf dry weight per tiller in autumn (Table 1). However, there being more roots per phytomer in autumn, mean dry weight of the individual roots (RDWi) of spring and autumn roots averaged across all phytomers was approximately equal (*p* = 0.748 in repeat measures ANOVA). The highest observed RDWi values were reached in both seasons after approximately 50 days of growth. The mean RDWi for roots of age ≥ 48 days (root age in phyllochron time-scale) in spring was 20.1 ± 1.19 mg, with these roots located at phytomer (Pr) 7–10 considering the youngest leaf (EL in Figure 1A) as the reference point. By contrast, in autumn, despite shoot size being much larger than in spring, mean RDWi of roots of age ≥ 48 days was 14.9 ± 0.84 mg, with these roots located at Pr11–16 (*p* = 0.033 for the phytomer position × season interaction in repeat measures ANOVA). An analysis with fine roots of diameter classes < 0.1 mm and 0.11–2.0 mm indicated that, compared to autumn roots, old spring roots produced much greater root length and surface area, and had a greater volume of fine roots (Figure S1). Root main axis length was 36.9 ± 1.04 cm averaged over 180 observations in spring and 26.3 ± 1.05 cm averaged over 192 observations in autumn with significant variations between two seasons (Table S2).

**Figure 2:**
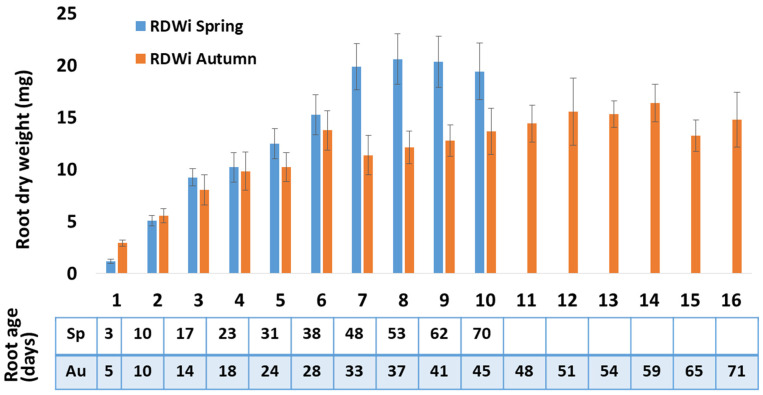
Individual root dry weight (RDWi) observed for Aberdart perennial ryegrass at successive root bearing phytomers in Spring and Autumn experiments. The number of roots per phytomer (Rp) is provided in Table S2. The panel below shows root age in spring (Sp) and autumn (Au) in days. Data are means for 30 and 16 plants in spring and autumn, respectively, whose roots were dissected by phytomer position. Vertical bars indicate the standard error of the mean value.

On average, spring Pr bore 1.87 roots, while autumn phytomers bore 2.64 roots per phytomer (Table S2). Across spring and autumn data sets, the mean number of roots per phytomer and the mean temperature recorded for the period when root initiation occurred at that phytomer (Table S2), were significantly correlated (r = 0.532; *p* = 0.005). Root dry matter deposition rate declined as roots aged, reaching zero approximately 50 days after root initiation (Figure 2, Table S2; [21]).

### 2.3. Ontogeny of Root Morphological Development

#### 2.3.1. Total Length, Surface Area and Volume of Individual Roots

**Figure 3:**
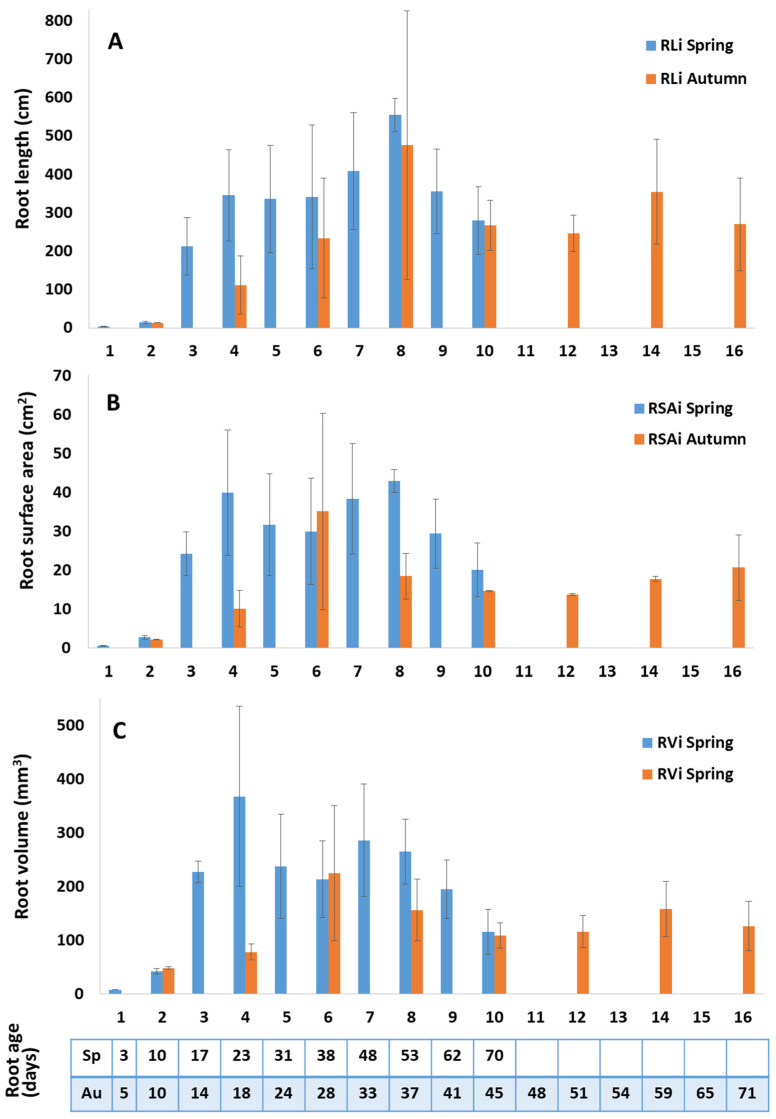
Individual root measures at successive root bearing phytomer positions (Pr) for Aberdart perennial ryegrass in the spring (Sp) and autumn seasons (Au); (**A**) total root length of individual roots (RLi), (**B**) root surface area of individual roots (RSAi) and (**C**) root volume of individual roots (RVi). The panel below shows root age in spring (Sp) and autumn (Au) in days. Data are from two plants in each season whose roots dissected by phytomer position were scanned in WinRhizo^®^ (Regent Instruments Inc., Quebec, Canada). Vertical bars indicate the standard error of the mean value.

Winrhizo scanning of roots of two plants in each season gave a picture of the ontological development of roots at successive phytomers from the combined effects of rate of root dry matter deposition and branching. Individual roots with their branches under the hydroponic growth conditions of this experiment, developed a total length of 500–600 cm, a total surface area of 35–45 cm^2^, and a volume of 250–350 mm^3^ (Figure 3A–C), with most of this growth occurring over the first 25–30 days (i.e., over Pr1–4 in spring and Pr1–6 in autumn experiments). Individual root volume showed an increasing trend in both seasons up to a root age of approximately 37 days of age (i.e., Pr5 in spring and Pr8 in autumn) and the phytomer age effects were confirmed as statistically significant (*p* = 0.028 for regression coefficient, Figure 3C).

A number of derived measures of root area development linked to branching or smaller diameter in roots of the same branching order were calculated for these data, including: mean root diameter (RD, mm; Table S1), number of root tips (Table S1), specific root length (SRL, cm mg^−1^), and ratios of length:volume (RL/RV, cm mm^−3^) and surface area:volume (RSA/RV, cm^2^ mm^−3^) (Figure S2). General features of this data are high variability between individual roots and a general pattern of root area and branching development (Figure S2) continuing after increase in individual root dry weight, total length, surface area, and volume had reached their maximum values.

#### 2.3.2. Phases of Root Development

**Figure 4:**
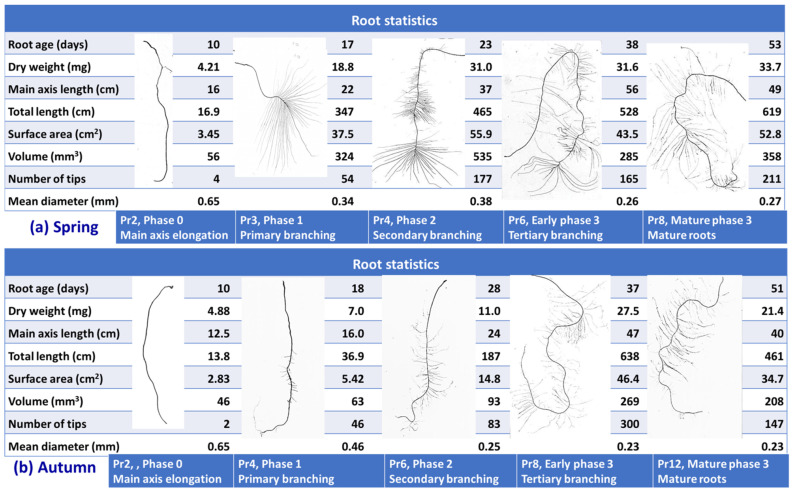
Individual WinRhizo^®^ scanned roots at different phytomer positions (Pr) along with root statistics showing succession of root development for Aberdart perennial ryegrass in (**a**) spring and (**b**) autumn seasons. Root phases were defined by branching order. Maturity of roots at the late phase 3 was determined by ongoing increase of specific root length without increasing dry matter deposition. Table S3 compares average values of root traits of Pr3-spring vs. Pr4-autumn and Pr8-spring vs. Pr12-autumn with statistical significance.

Visual scoring identified tertiary order branching of older roots in spring and some quaternary order branching of older roots in autumn. Based on development of successive orders of root branches, four phases of root development were identified, as illustrated by representative scans (Figure 4): Phase 0, main axis elongation with little branching; Phase 1, ongoing main axis elongation and root mass deposition with substantial primary branching; Phase 2, main axis elongation and dry matter deposition rates slowing and secondary branches appearing; and Phase 3, little or no main axis elongation or dry matter deposition occurring with tertiary and quaternary branches appearing and evidence of simultaneous root growth and localized senescence. In spring, substantive first order root branching was initially observed in roots located at Pr2 and Pr3, approximately 10 days after root appearance, while secondary and tertiary order root branching was first observed at Pr4 and Pr6, 20 days and 35 days, respectively, after root appearance (Figure 4). In autumn, first order lateral branches first appeared in numbers at roots located between Pr2 and Pr4, from approximately 10 days after root appearance, while secondary order root branching occurred on roots located between Pr4 and Pr6 around 18 days after root appearance and tertiary order root branching occurred between Pr6 and Pr8 on roots around 30 days of age and quaternary root branching occurred on roots aged 50 days or more (Figure 4).

## 3. Discussion

### 3.1. Ontogeny of Root Developemnt

Examples of shoot axis observation to recover historic information on plant development include studies of *Poa pratensis* [22], and *Carex bigelowii* [23]. Matthew and Kemball [12] adapted this technique to dissect an age-series of roots from perennial ryegrass tiller axes. In this study we have added the innovation of monitoring leaf appearance in perennial ryegrass tillers under study for a 90-day period prior to destructive harvest. Since phytomers producing successive leaves initiate roots to become root-bearing phytomers upon senescence of the leaf they bore, recording the date of death of each leaf allowed the date of initiation to be inferred for individual roots. In this way we were able to reconstruct root development curves for the dissected tillers over the 90-day period of leaf observation.

The main features of the root development process indicated by the data from these experiments were, (i) in both seasons, the main axis elongated after initiation at an average rate of approximately 1.3 cm d^−1^ with little or no branching in the first 10 days (Phase 0); (ii) over days 10–25, active main root axis elongation continued with the appearance of primary and secondary branches (Phases 1 and 2); and (iii) between days 30–40, main axis elongation ceased and tertiary root branching began to occur (Phase 3). In phase 3, there was typically continued increase in specific root length and the ratio of RSA:root mass (or volume), and sometimes appearance of quaternary branches, but no trend of increase in root mass, volume, or numbers of tips, suggesting simultaneous senescence and ongoing ramification in different branches of the same roots.

In younger phytomers, the addition of <1.0 mg dry matter Pr^−1^ d^−1^ allowed the construction of approximately 50 mm^2^ RSA, not considering the impact of root hairs on RSA. The rate of dry matter deposition decreased by approximately 50% at Pr3–4 when first order of branching commenced and further decreased to one-quarter its initial rate at Pr6–7 when secondary and tertiary order root branching occurred. The decline in dry matter deposition with root age was shown [21] to be mainly related to increased C costs of maintenance respiration by larger, older roots, rather than to reduced photosynthetic C supply, suggested elsewhere [12]. At the older phytomer positions, secondary and tertiary roots made up a greater fraction of the root system, leading to a reduction in the mean root diameter and that process is also associated with lower dry matter deposition rate at each phytomer (DMDp) and C requirement for root construction.

The maximum mean root axis length (RAL) measured in these experiments was 42 cm (with some individual roots reaching c. 70 cm); however, in some other studies perennial ryegrass roots can grow to more than 1.0 m in length [24]. Previous data indicated that growing medium and moisture stress affect root axis length [12,25,26]. The reason for the shorter RAL in this study is not known but the removal of daughter tillers may be important as previous studies have demonstrated that daughter tillers can contribute significantly to the root C budget in several grass species [27,28].

For individual phytomers on the tiller axis, this ontogenetic root development sequence captured in these data can be conceptualized as continuous with and paralleling the leaf development and senescence sequence [29] which is typically of 3–4 phyllochrons duration in perennial ryegrass. In this study, no case of complete senescence of an individual root was recorded within the observation period of just over 80 days. For a tiller axis harvested from a field sward in spring, one study [30] reported root death about 12 phytomers below root initiation, which would suggest that Phase 3 roots at Pr11 in the Spring experiment might have been about to undergo senescence. It has been reported that the survival of individual perennial ryegrass roots ranges from several months up to one year [9,31,32]. On the other hand, the longevity of lateral root branches may be less than 21 days [33]. These contrasting observations on the longevity of axial roots compared with their branches are consistent with evidence in the present study suggesting simultaneous growth and death of finer branches of older roots.

### 3.2. Hypothesized Seasonal Shift in C Supply to Roots

The basis of the postulated architectural signal resulting in enhanced C-supply to spring roots compared to autumn roots effectively stems from seasonally driven changes in the ratio between the rate of new leaf formation and the rate of new root formation. These shifts are a result of leaf appearance happening earlier in the life of a phytomer, with root appearance occurring on the same phytomer several weeks later. Thus, changes in the rate of phytomer development will first manifest in the leaf area, with a lag period before being mirrored by changes in root initiation. Although not in itself definitive, we observed on average 11 phytomers with roots initiated over 83 days in spring and 18 phytomers with roots initiated over 80 days in autumn, a 70% higher potential root appearance rate in autumn.

In addition, values for number of roots per phytomer were higher in warmer temperatures of autumn, averaging 1.89 and 2.63 in spring and autumn, respectively (Table S1, [3]). This observation raises a question for further investigation, whether this increase in Rp is a generic temperature response of perennial ryegrass or grasses in general. If so, there are interesting ecological implications for seasonality of root-system behaviour. Taking the data from this experiment, the increased number of Rp in autumn would therefore further reduce the substrate available to individual roots per phytomer by a ratio of 1.89:2.63, so enhancing the root growth constraint (compared to spring) of the phyllochron being longer than the rhizochron in autumn. In this example, the greater number of root bearing phytomers in 90 days in spring than autumn, and the increased number of roots per phytomer in autumn combine multiplicatively to an expectation of initiated root numbers for the observation period being 2.39× greater per tiller in autumn than in spring. Given the leaf weight and leaf number per tiller in Table 1 we can estimate mean lamina weights of 61.5 mg in spring and 124.5 mg in autumn, and mean RDWi of 13.1 mg in spring and 12.4 mg in autumn (mean of all Pr of Figure 2), indicating respective root:lamina weight ratios of 21.3% in spring and 9.9% in autumn, a factor of 2.15× and approximately consistent with the proportional difference in numbers of roots initiated (see Supplementary Data File S2). The conclusions of these calculations that individual autumn roots tend to be smaller than individual spring roots are supported by the statistically significant season × phytomer age interaction, stemming from the comparatively longer primary branches of younger spring roots, and the heavier final weights of spring than autumn roots aged greater than 50 days. The results in this case show the numerical effect of the seasonal shift in rhizochron and phyllochron on number of root bearing phytomers and an effect on mature root dry weight consistent with increased competition between individual roots for C supply in autumn compared to spring. Matthew et al. [30] also found features in field data for new root deposition consistent with seasonal change in rhizochron:phyllochron ratio, and one corollary for future investigation is that such an effect would facilitate seasonal specialization of roots, with spring roots tending to penetrate to deeper soil layers to maintain summer moisture supply, while autumn-formed roots would tend to occupy soil strata nearer the surface, which may be advantageous in winter when deeper soil layers may become waterlogged and hypoxic. Deeper penetration of spring-formed roots would be further facilitated by cessation of new root initiation in dry summer conditions [30,34,35], in this way avoiding diversion of substrate supply to later-formed young roots and providing for ongoing substrate supply to earlier-formed roots in drought conditions. In this study, no reproductive tiller was observed in either the spring or autumn experiment.

### 3.3. Branching as a Mechanism for Surface Area Development

The root branching process is continuous and the commencement of one branching order does not require the termination of another. Root branching produces sequentially finer roots, which greatly reduces construction costs [21]. A linear decrease in dry matter deposition rate at older phytomers is probably associated with reduced C availability for growth [12,21]. Root branching may therefore be an adaptive strategy, allowing plants to reduce construction cost per unit soil explored as roots age and C supply becomes limiting.

During the main root axis elongation phase (Phase 0 in Figure 4) root diameter ranged between 0.6 to 0.9 mm (Figure 4). These main root axes added around 5 mm^3^ of volume per day (Figure 4). Although comparatively expensive in terms of dry matter investment, comparatively greater diameter would provide increased mechanical strength to penetrate to deeper soil layers, and can accommodate more primary branches, potentially providing better persistence to grazing and facilitating a more efficient vascular distribution network to facilitate rapid water flux, connecting to laterals and finer branches [36]. Root thickness and maximum root length traits have been confirmed to co-locate in quantitative trait locus studies [37].

Root diameters of the primary branches are 50–33% of those of the main axis. At the commencement of first order root branching root surface area and root volume exhibit exponential increases (Figure 4, Table S1). A first order lateral root with ^1^/_x_^th^ of the root diameter of its main axis requires only ^1^/x^2^ times the dry matter to build a unit length of root and therefore allowing production of _X_^2^ fold RL at the same construction cost. A 5–10 times higher RL addition occurred at first order of branching compared to main axis elongation phase. Another theoretical implication is that when root branches are of smaller diameter than their parent root is that a greater number of branches can be accommodated on the parent root, facilitating proliferation. It can be shown that when the root diameter of primary roots reduced to ^1^/_3_ of the main axis then 9× more branch roots can be accommodated per unit RL of the parent root, so increasing the potential soil volumes explored or the root density per unit soil volume [38,39].

These same principles also apply to generation of secondary and tertiary order lateral roots. The root surface area greatly increases during phase one and two, with presumed importance in terms of nutrient and water uptake [40]. Secondary root branches generally originate from the pericycle of the vascular cylinder. The diameter reduction at this phase was estimated to be approximately ^3^/_4_ of the primary branches requiring a dry matter deposition approximately ^9^/_16_ of the primary branches per unit RL increase. Spring plants produced a greater length, surface area and volume by producing greater finer roots of <0.1 mm diameter compared to autumn plants and those finer lateral roots largely contribute to secondary and tertiary lateral branches and thus increase total root surface area per tiller [38,41,42,43]. For tertiary lateral roots, given a similar rate of photosynthetic carbohydrate supply to phase 0, approximately 36–64 times higher RL production is theoretically possible. Clearly, the continued branching and ramification of older roots would allow additional surface area in mature roots to be constructed with fine diameter roots at comparatively low dry matter cost [44], which would be advantageous in conditions of C-supply constraint.

Quaternary branching was rarely seen in the spring experiment while in the autumn experiment this level of branching was observed at Pr14 (Figure 4). The roots of quaternary branches are < 0.1mm diameter in general. The dry matter addition at this phase is estimated to be very low. It has been observed that root branches can display partial disintegration, loss of cortical tissue and thus reduced root volume in this phase. If C is transported from those disintegrating old tissues to generate new finer branches and root hairs then uptake activity of the roots might be continued. The contribution of root hairs to RSA was not measured in this study but would be major. For example, in wheat root hairs were calculated to provide 94.6% of estimated RSA at Pr4 [39].

### 3.4. Extrapolation of Results to Field Swards

**Figure 5:**
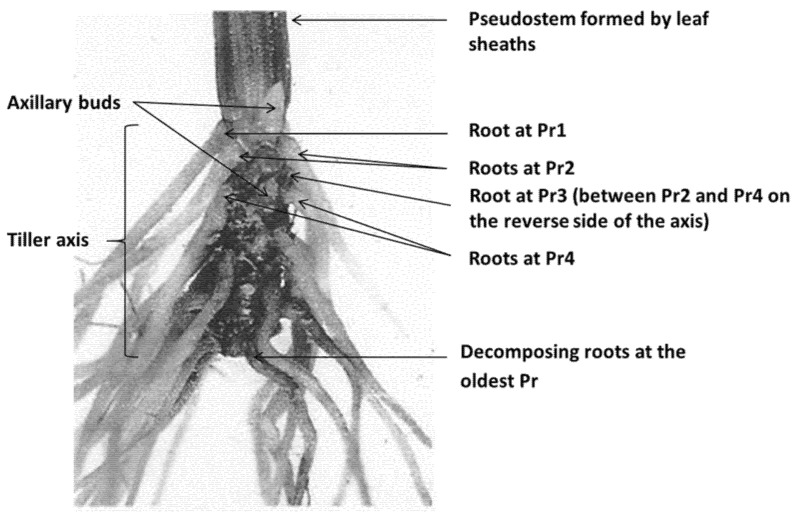
Axis of a perennial ryegrass tiller showing white younger roots at root bearing phytomers (Pr), Pr1–Pr4 and decay and decomposition of roots at the oldest Pr. The root bearing phytomers collectively make up the tiller axis.

The experiments were necessarily conducted in hydroponic culture to facilitate dissection of single roots without undue damage, but this raises the question of how the present results would extrapolate to field swards. Figure 5 (below) depicts the tiller axis of a plant grown in soil, and the structural organization was considered by the authors to be virtually identical to that of the hydroponically grown plants. There are few published studies of single root counts in field swards, but in one New Zealand study [45] minirhizotron counts of new roots appearing, consistently showed a strong autumn peak in each of five perennial ryegrass varieties over two seasons, while in two studies counting new nodal roots appearing at the tiller axis [10,46], a late winter or early spring peak was observed. Further research could be initiated to determine if these superficially contradictory field results can be explained by the seasonal differences in root formation identified in the present study.

## 4. Materials and Methods

### 4.1. Plant Culture

*Lolium perenne* plants were grown in a hydroponic culture unit in a glasshouse at the Plant Growth Unit, Massey University, Palmerston North, New Zealand (latitude 40°19′ S, longitude 174°46′ E, altitude 25 m amsl, Figure S3A,B). Two separate experiments were conducted. In spring, plants were grown from 1 July 2008 to 28 September 2008 and in autumn from 3 March 2009 to 31 May 2009 referred to as spring and autumn experiments, respectively [21]. In both experiments, hourly temperature data inside the glasshouse were recorded using a micrologger (Model Z, HortPlus.com (13 August 2021)). Summaries of the temperature data were presented by Robin [19] (Supplementary Data File S3).

To provide populations of plants for each experiment, plants of ten genotypes of perennial ryegrass cultivar Aberdart were chosen and adult tillers divided out from each plant to provide three clonal replicates of each genotype for transplanting. Any small daughter tillers attached to tillers at transplanting were removed. The length of tillers from pseudostem base to leaf tip at transplanting was 20–25 cm. The transplanted tillers were grown hydroponically in modified Hoaglands solution [20,39] for 84 days before commencing destructive harvesting. Leaf elongation rate (LER) in all plants was calculated as measure of final leaf length (cm) divided by the leaf elongation duration (days), converted to LER in growing degree days (mm °C^−1^ d^−1^). Harvesting took place over eight consecutive days. During the growing period only the parent tiller and the two first-formed daughter tillers were allowed to develop and all other daughter tillers were removed on appearance (Figure S3C). The hydroponic culture had an aerated recirculating system. The details of hydroponic culture, composition of nutrient solution and plant management are described by Robin et al. [20,21].

### 4.2. Root Harvest

Three clonal replicates from ten genotypes in spring and two clonal replicates of eight genotypes in autumn were harvested. Roots from the individual phytomer positions on the tiller axis were excised in sequence from youngest to oldest using a scalpel and 15× magnification binocular microscope (Figure S3D and Figure 5). Excised roots were carefully teased apart from the other roots under water and stored as described below for analysis. The number of root bearing phytomers per tiller, number of roots per phytomer (Rp) and total number of roots per tiller were recorded at harvest for all plants, and the length of each root main axis (RAL) was measured in cm for eight plants from eight genotypes of each cultivar.

### 4.3. Root Age Determination

The number of phytomers between the youngest leaf position and the first root appearance at the tiller axis was noted for each tiller (d value in Robin et al. [20]). The time interval in phyllochrons was noted. The root bearing phytomers were numbered consecutively beginning with the phytomer with the youngest roots as a reference point (Pr1). The age of the roots at a particular Pr was estimated based on an assumption of steady state progression of phytomers on the tiller axis from leaf to root production [11]. That is, the root at Pr1 was assumed to be the same age as the youngest leaf. The age of roots at successive phytomers below Pr1 was estimated by adding the phyllochron for the leaf observed earlier in the experiment at that particular phytomer, to the age of the root at the phytomer above.

### 4.4. Root Measurements

A total of 571 individual roots were isolated from 301 phytomers from 30 tillers in spring and 766 individual roots from 288 phytomers from 16 tillers in autumn. Scanning all roots for detailed morphological data using WinRHIZO^®^ was not feasible due to time constraints. Consequently, root dry weights were recorded for all harvested roots, and scanned images were made of subsets of roots. Two randomly selected plants of two different genotypes for each cultivar in each season were scanned as described below.

### 4.5. Root Preservation and Processing

In the autumn experiment, roots selected for scanning and WinRHIZO^®^ analysis were preserved in 70% ethanol. Remaining roots were dried in a forced air draft oven at 60 °C for two days, and the root dry weights from individual phytomers (RDW_p_) were determined. Roots from the spring experiment were preserved in 70% ethanol and stored until the samples could be processed.

### 4.6. Root Scanning Using WinRHIZO^®^ Software

In spring, roots from all phytomers and in autumn, the roots from even-numbered phytomers of the two selected plants were scanned (Figure S3E). A total of 37 roots from 22 phytomers were scanned in spring and 22 roots from 16 phytomers in autumn at the AgResearch, Ruakura Research Centre in Hamilton, New Zealand using WinRHIZO^®^ software (Regent Instruments Inc., Quebec, Canada). Individual roots were placed in a clear acrylic tray and spread out using a needle so there were no overlapping roots. The roots were scanned on a flatbed scanner (STD1600+, Regent Instruments Inc., Quebec, QC, Canada) at 210 dpi resolution. Values for root length (RL, cm) which included main axis and branches, surface area (RSA, cm^2^), volume (RV, cm^3^), and mean diameter (RD, mm) were obtained. A set of old roots from both spring and autumn grown plants were also scanned to investigate the behaviour of fine root statistics. The total number of tips for individual roots (RT_i_) was counted using Videopro 32^®^ software (Leading Edge Pty Ltd., Adelaide, SA 5042, Australia).

### 4.7. Root Data Derivation

#### 4.7.1. Root Dry Weight Correction

The roots kept in ethanol for scanning were rinsed in water and oven dried at 60 °C for 48 h. Dry weights were corrected by for an assumed 22.4% weight loss of alcohol soluble sugars during storage [47].

#### 4.7.2. Root Dry Weight of the Individual Roots (RDWi)

Root dry weights were recorded for the individual phytomers (RDWp). From those data the mean dry weight of the individual roots (RDWi) was obtained as RDWp divided by number of roots per phytomer (Rp).

#### 4.7.3. Dry Matter Deposition Rate at Each Phytomer

DMDp (mg Pr^−1^ d^−1^) was estimated from the difference in root dry weight between that Pr and successively older Pr, assuming the age difference of roots at the successive phytomers was equal to the phyllochron at that Pr.

#### 4.7.4. Specific Root Length, Surface Area, and Volume

The specific root length (SRL, cm mg^−1^), specific root surface area (SRSA, cm^2^ mg^−1^) and specific root volume (SRV, mm^3^ mg^−1^) of the scanned roots were calculated as the ratio between the respective individual root data for each trait and dry weights for those particular individual roots (RDWi) at the different phytomer positions.

### 4.8. Visual Scoring for the Root Branching Orders

The order of root branching (primary, secondary, tertiary, etc.) at the different phytomers in succession moving down the tiller axis was scored by visual assessment of the root images. To obtain a branching score-sheet for all scanned roots, phytomer positions were numbered from Pr1 to Pr ‘n’ (where n is the oldest phytomer) on the *X*-axis and the order of root branching was scored on the *Y*-axis.

### 4.9. Root Length:Volume and Surface Area:Volume Ratios

As a measure of the extent to which branching and associated mean diameter reduction in older roots enabled greater soil exploration and absorptive area per unit of RDW, the ratios RL:RV and RSA:RV were derived. Dimension corrections of these ratios often used in plant allometry to separate effects of size and shape were not adopted in this study because grass roots at each successive branching order were considered to be cylindrical in form and of uniform diameter along their length.

### 4.10. Statistical Analysis

Data were analysed using the ‘repeat measures’ procedure in SAS version 9.4 (SAS Institute, Cary, NC, USA) Proc GLM [48,49] to test for season, phytomer position and season x phytomer position interactions. Data for different phytomer positions on the same tiller axis were treated as repeat measures. For repeat measures analysis spring and autumn data were aged matched by averaging autumn data for two or three adjacent autumn phytomers where relevant to produce a mean value for a root of the same age as the corresponding spring root. Where data properties indicated, data were log-transformed (Table S1, Figure S2).

To test the effect of season, genotypes within season and phytomer positions within season a user-defined ANOVA model was applied (see Table S2). To test the effect of season, genotypes within season was used as an error term, and to test the effect of genotype and phytomer position, clonal replicates within genotype and season was used as the error term. A one-way ANOVA was performed to test statistical significance between two phytomers of similar age (see Table S3) and also fine roots of individual old roots from two seasons (see Figure S1).

A Pearson correlation analysis was conducted between roots per node and mean temperature for the period when root initiation occurred at that phytomer. A linear regression coefficient between root volume and root age up to 37 days was obtained.

## 5. Conclusions

In conclusion, the data presented here have characterized perennial ryegrass root development over time in spring (with increasing day-length and temperature) and autumn (with decreasing day-length and temperature) using a phyllochron time scale. Surprisingly, while such data for leaf development at successive phytomer positions on the tiller axis have been available for over 40 years (e.g., Davies [50]) the corresponding root data have been unavailable. Producing those data here has been possible with a novel technique of recording leaf appearance events over several months in order to infer the date when individual phytomers on the tiller axis would have initiated their roots, and growing plants in hydroponic culture to facilitate dissection of individual roots from the bulk root mass. The present study splits root development into four phases based on branching order. Unbranched, rapidly elongating main axes (phase 0) were observed at phytomer positions 1 and 2, primary branching (phase 1) occurred at positions 3 and 4, secondary branching (phase 2) followed at positions 5 and 6 and then tertiary branching (phase three). Rapid development of root length and surface area occurred during phases 1 and 2 as branching began and progressed. Maximum root dry weight was attained after 7 phyllochrons (50 d) in spring and 6 phyllochrons (30 d) in autumn (Figure 2). Thereafter, as roots matured in phase 3, there was little increase in root length or root area (Figure 4A,B), but ongoing increase in root statistics such as specific root length or area:volume ratio (Figure S2A,B), from which we infer ongoing proliferation of fine branches with concurrent senescence. Strong seasonal and phytomer age effects and a season × phytomer age interaction were found, with spring plants having fewer root-bearing phytomers, but achieving larger maximum root masses. This insight will provide concepts and base data for realistic mechanistic modelling of grass root system behaviour and further investigation of the ecological and functional implications of the differing behaviours of spring and autumn roots.

## Figures and Tables

**Table 1:** Differences in plant shoot development in spring and autumn experiments. Statistical difference between spring and autumn was tested using one-way ANOVA.

Variables	Spring	Autumn	*p* Value
Leaf dry weight per tiller (mg) ^a^	498.1 ± 27.8	1515 ± 60	<0.01
Number of live leaves per tiller ^a^	8.1 ± 0.15	12.25 ± 0.47	<0.01
Range of leaf elongation rates (mm °C.d^−1^)	1.4 to 2.4	1.7 to 2.7	<0.01

^a^ Data represent the average of 30 and 16 dissected individual tillers, respectively in spring and autumn seasons.

## Data Availability

All data included as Supplementary Materials.

## Supplementary Materials

The following are available online at https://www.mdpi.com/article/10.3390/plants10081677/s1, Table S1. Analysis of variance of root variables following general linear model to show seasonal and phytomer position within season variations in individual roots measures. RDW_i_, root dry weight of individual roots; RL_i_, RSA_i_ and RV_i_–WinRhizo root length, surface area, and volume of the individual scanned roots; SRL, specific root length (cm mg^−1^); RT_i_, number of root tips per root; RD, root diameter; df, degrees of freedom; MS, mean squares. Table S2. Root measures at the successively more developed and older phytomer positions (Pr) on the tiller axis in spring and autumn seasons as indicated by number of roots per phytomer (Rp), root dry weight per phytomer (RDWp), dry matter deposition rate (mg Pr^−1^ d^−1^), main root axis length (RAL), mean root diameter (RD), and number of root tips per root (RTi). SE, standard error of mean, when presented as percent is for log transformed data. Table S3. Measures of individual roots of the scanned roots of similar age during primary root branching (Phase 1) and tertiary root branching (Phase 3) in spring and autumn grown perennial ryegrass plants. Each data represents average ± SE of two scanned roots at each root bearing phytomer (Pr) position. Bold fonts represent statistically significant variation between spring versus autumn roots (*p* < 0.05)^1^. Figure S1. Variation in root length, surface area and volume of fine roots in old roots of >48 days with diameter <0.1 mm and <0.11–0.2 mm diameter between spring and autumn seasons. L, length; SA, surface area and V, volume. Figure S2. Specific root length (A, SRL, cm mg^−1^), ratio between root length and volume (B), surface area and volume (C) of Aberdart perennial ryegrass cultivars in spring and autumn experiments. Dotted fitted lines indicate quadratic curves. Vertical bars indicate standard error of mean. Two-way analysis of variance was conducted to test season and phytomer with season interaction for the repeat measures. Figure S3. Experimentation and data collection with perennial ryegrass in hydronic plant culture unit. (A) experimental set-up in hydroponic culture, (B) plants ready for destructive harvest, (C) a single plant before dissecting roots from the tiller axis, (D) dissected roots from a single plant, and (E) scanning roots using WinRhizo software. Supplementary Data File S1. Data for root dry weight, root axis length and morphological traits of lateral roots obtained through root scanning, Supplementary Data Files S2. Calculation for seasonal difference in the rhizochron:phyllochron ratio, Supplementary Data File S3. Daily maximum and minimum temperatures, degree days, sunshine hours in New Zealand during spring and autumn experiments.
