# Supplementary material for: Time Course of Root Axis Elongation and Lateral Root Formation in Perennial Ryegrass (Lolium perenne L.)
Source: Plants (Basel). 2021 Aug 15;10(8):1677. doi: 10.3390/plants10081677 (PMC8399834; doi:10.3390/plants10081677)
Supplement: Supplementary file 1 [file plants-10-01677-s001.zip › Supplementary data-Proof.pdf]

Supplementary data

Table S1. Analysis of variance of root variables following repeat measures ANOVA in SAS (version 9.4) to study variations in individual roots measures for season, phytomer and phytomer-season interactions. RDW_i, root dry weight of individual roots; RL_i, RSA_i and RV_i - WinRhizo root length, surface area and volume of the individual scanned roots; SRL, specific root length (cm mg^{-1}); RT_i, number of root tips per root; RD, root diameter; df, degrees of freedom; MS, mean squares.

Trait	No. of observations	Data presented in	Sources of Variation	df	MS	F Statistic	*p* Value	Sources of Variation	df	MS	F Statistic	*p* Value
**RDW_p**	Spring = 300	Fig. 2	Pr	8	16.8	26.12	<0.001	Season	1	15.7	8.59	0.006
	Autumn = 256		Pr*Season	8	1.42	2.22	0.026	Error	41	1.82		
			Error	352	0.64							
**RDW_i**	Spring = 300	Fig. 2	Pr	8	13.8	43.04	<0.001	Season	1	0.088	0.10	0.748
	Autumn = 256		Pr*Season	8	0.682	2.13	0.03	Error	44	0.85		
			Error	352	0.321							
**RL_i**	Spring = 22	Fig. 3	Pr	7	4.83	19.87	<0.001	Season	1	1.00	0.47	0.56
**(log-transformed)**	Autumn = 16		Pr*Season	7	0.105	0.43	0.865	Error	2	2.12		
			Error	14	0.243							
**RSA_i**	Spring = 22	Fig. 3	Pr	7	2.845	12.58	<0.001	Season	1	3.098	3.95	0.185
**(log-transformed)**	Autumn = 16		Pr*Season	7	0.09	0.40	0.885	Error	2	0.784		
			Error	14	0.226							
**RV_i**	Spring = 22	Fig. 3	Pr	7	1.051	7.91	<0.001	Season	1	2.44	2.75	0.239
**(log-transformed)**	Autumn = 16		Pr*Season	7	0.128	0.96	0.492	Error	2	0.889		
			Error	14	0.132							
**SRL**	Spring = 22	Fig. S1	Pr	7	1.77	19.33	<0.001	Season	1	0.103	0.44	0.57
**(log-transformed)**	Autumn = 16		Pr*Season	7	0.168	1.83	0.167	Error	2	0.233		
			Error	14	0.091							
**RL/RV**	Spring = 22	Fig. S1	Pr	7	1.589	16.82	<0.001	Season	1	0.319	1.18	0.39
	Autumn = 16		Pr*Season	7	0.048	0.52	0.806	Error	2	0.271		
			Error	14	0.094							
**RSA/RV**	Spring = 22	Fig. S1	Pr	7	0.45	7.05	0.001	Season	1	0.035	3.09	0.22
	Autumn = 16		Pr*Season	7	0.041	0.65	0.65	Error	2	0.011		
			Error	14	0.063							
**RT_i**	Spring = 22	Table S1	Pr	7	7.66	38.69	<0.001	Season	1	0.059	0.09	0.797
**(log-transformed)**	Autumn = 16		Pr*Season	7	0.06	0.30	0.94	Error	2	0.699		
			Error	14	0.198							
**RD**	Spring = 22	Table S1	Pr	7	0.439	17.37	<0.001	Season	1	0.086	0.83	0.459
**(log-transformed)**	Autumn = 16		Pr*Season	7	0.019	0.76	0.628	Error	2	0.104		
			Error	14	0.025							

Table S2. Root measures at the successively more developed and older phytomer positions (Pr) on the tiller axis in spring and autumn seasons as indicated by number of roots per phytomer (Rp), root dry weight per phytomer (RDWp in mg), dry matter deposition rate (DMDp in mg Pr^{-1} d^{-1}), main root axis length (RAL in cm), mean root diameter (RD in mm) and number of root tips per root (RTi). Tm, mean temperatures in glasshouse during the phyllochron when each phytomer first became root bearing; SE, standard error of mean, when presented as % is for log transformed data.

				Spring					Autumn							
Pr	Age (days)	Rp	RDWp	DMDp	RAL	RD	RTi	Tm	Age	Rp	RDWp	DMDp	RAL	RD	RTi	Tm
1	3	2.59	3.6	1.07	3.72	0.66	3.00	12.5	5	2.56	7.7	0.72	1.88			22.5
2	10	2.17	11.2	0.82	13.2	0.69	2.17	9.5	10	2.19	13.3	0.66	12.6	0.72	3.00	21.0
3	17	1.86	16.5	0.72	18.7	0.48	55.8	12.5	14	2.31	19.1	0.82	16.4			23.5
4	23	1.83	16.3	0.62	21.4	0.36	163	9.0	18	2.75	26.4	0.72	23.0	0.38	64.5	18.5
5	31	1.69	20.3	0.52	26.3	0.30	116	13.3	24	2.81	26.8	0.63	23.4			20.8
6	38	1.66	25.4	0.37	33.0	0.30	140	10.8	28	2.94	37.9	0.53	26.0	0.33	265	17.0
7	46	1.93	32.6	0.32	41.4	0.30	136	14.3	33	2.63	29.4	0.54	22.0			26.5
8	53	1.90	43.5	0.17	42.0	0.22	208	13.5	37	2.94	37.0	0.34	25.3	0.29	184	14.8
9	62	1.76	35.9	0.09	40.9	0.20	133	12.8	41	2.88	41.2	0.24	16.3			20.0
10	70	1.65	30.2	0.02	38.3	0.23	120	17.5	45	2.56	37.3	0.18	27.3	0.32	189	18.0
11	83	1.54	21.6		31.6	0.21	180		48	2.50	37.0	0.05	25.0			15.8
12									51	2.25	37.7		28.0	0.28	135	20.0
13									54	2.50	39.1		33.7			23.0
14									59	2.47	40.1		29.5	0.27	171	16.5
15									65	1.93	23.8		31.0			13.3
16									71	2.43	31.9		31.0	0.22	120	18.3
17									75	2.75	44.7		18.8			
18									80	4.09	63.1			0.22	146	
Total $tiller^{-1}$		20.6	257				1260			47.5	594				2560	
SE [a]	1.2%	0.055	7.2%	11.4%	7.25%	7.7%	35%		1.3%	0.084	5.2%	9.7%	7.25%	9.4%	45%	
p(season) [b]	<0.001	<0.001	0.023	0.184	<0.001											
P(genotype)	0.003	0.074	0.032	0.99	<0.001											
p(Pr)	<0.001	0.924	0.003	<0.001	<0.001											

[a] Since most of root traits except Rp had wider variation between younger and older roots the data were log transformed before ANOVA, and standard errors from ANOVA of log data were back transformed to obtain standard errors (SE) as ratios of the original mean values

[b] To test the effect of season, genotypes within season and phytomer positions within season a user-defined ANOVA model was applied. To test the effect of season, genotypes within season was used as an error term, and to test the effect of genotype and phytomer position, clonal replicates within genotype and season was used as the error term.

Table S3. Measures of individual roots of the scanned roots of similar age during primary root branching (Phase 1) and tertiary root branching (Phase 3) in spring and autumn grown perennial ryegrass plants. Each data represents average ± SE of two scanned roots at each root bearing phytomer (Pr) position. Bold fonts represent statistically significant variation between spring versus autumn roots ($p<0.05$)[1].

	Primary branching phase		Tertiary branching phase	
**Variables**	**Spring**	**Autumn**	**Spring**	**Autumn**
Phytomer position	Pr3	Pr4	Pr8	Pr12
Root age (days)	17	18	53	51
Dry weight (mg)	16.3 ± 2.5	21.8 ± 4.8	27.9 ± 5.8	16.2 ± 5.2
Main axis length (cm)	18.7 ± 3.3	42.2 ± 6.2	13.3 ± 4.0	34.3 ± 5.8
Total length (cm)	212.3 ± 74.8	112.1 ± 75.3	**554.8 ± 43.4**	**246.7 ± 47.8**
Surface area (cm^2)	24.2 ± 5.6	10.1 ± 4.7	**42.9 ± 3.0**	**13.7 ± 0.2**
Volume (mm^3)	**227 ± 20**	**78 ± 15**	265 ± 60	116 ± 30
Number of tips	82 ± 30	65 ± 18	208 ± 8.0	135 ± 28
Mean diameter (mm)	0.41 ± 0.09	0.36 ± 0.11	0.24 ± 0.02	0.25 ± 0.01
Specific Surface Area (cm^2 mg^{-1})	**1.47 ± 0.11**	**0.59 ± 0.14**	1.55 ± 0.07	0.94 ± 0.29

[1] Statistical significant difference of Pr3-spring versus Pr4-autumn and Pr8-spring versus Pr8-autumn was tested using one-way ANOVA command.

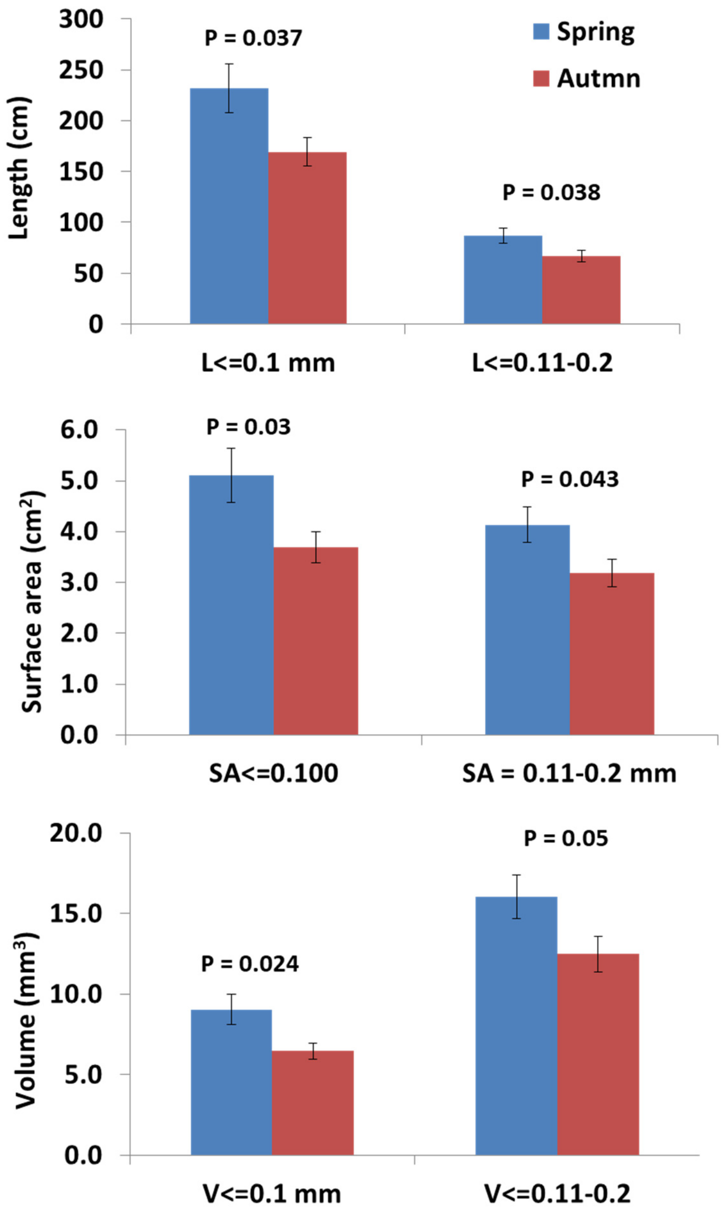

Figure S1. Variation in root length, surface area and volume of fine roots of individual old roots of >48 days with diameter <0.1 mm and <0.11– 0.2mm diameter between spring and autumn seasons. L, length; SA, surface area and V, volume. P value was determined based on one-way ANOVA in Minitab. The number of observations (n) was 21 and 24, respectively, in spring and autumn.

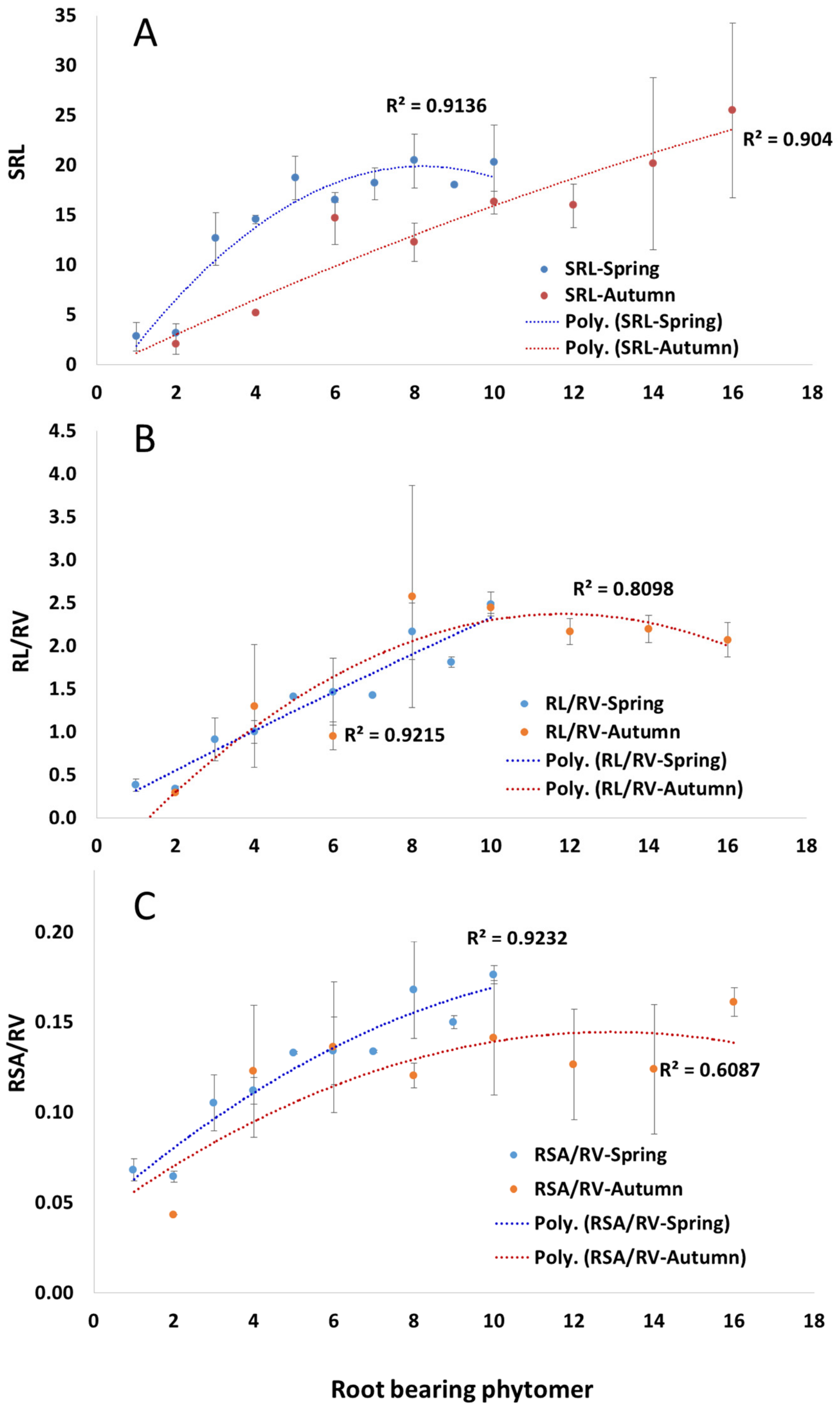

Figure S2. Specific root length (SRL, cm mg^{-1}) (A)**,** ratio between root length and volume (cm mm^{-3}) (B), surface area and volume (cm^2 mm^{-3}) (C) of Aberdart perennial ryegrass cultivars in spring and autumn experiments. Dotted fitted lines indicate quadratic curves. Vertical bars indicate standard error of mean. Two-way analysis of variance was conducted to test season and phytomer with season interaction for the repeat measures.

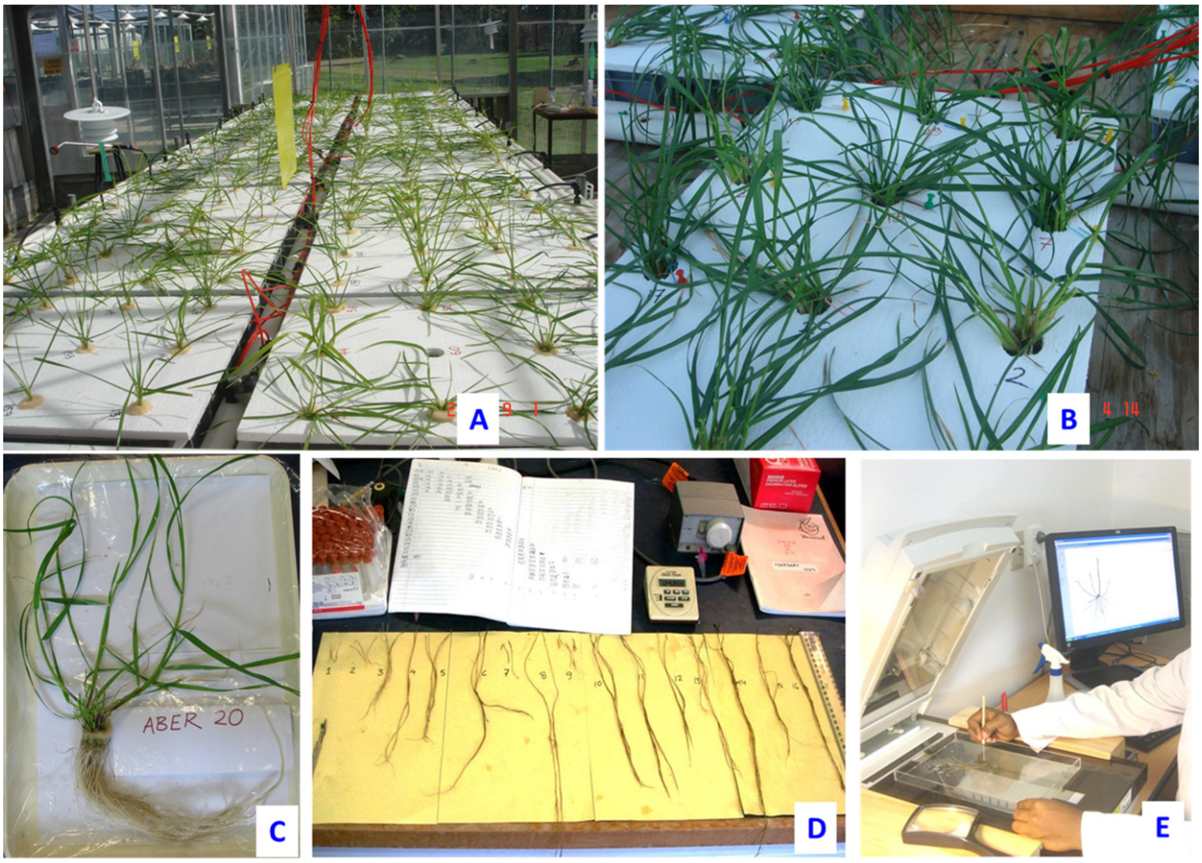

Figure S3. Experimentation and data collection with perennial ryegarss in hydropnic plant culture unit. A) experimental set-up in hydroponic culture, B) plants ready for destructive harvest, C) a single plant before dissecting roots from the tiller axis, D) dissected roots from a single plant and E) scanning roots using WinRhizo software.
